# Manchester Hospital War Work

**Published:** 1915-06-12

**Authors:** 


### MANCHESTER HOSPITAL WAR WORK.

MANCHESTER to-day is indeed a city of action; of work so well organised and so quietly accomplished that it is difficult to realise all that is being done for the wounded men who arrive almost daily.

About thirty thousand soldiers have received treatment in the last nine months, and everywhere there is the feeling that no effort is too great to make for the relief of these men. The first military hospital had six hundred beds, a number soon found inadequate, and auxiliary hospitals were opened. The work in those early days was greater for all concerned than now, for so many have given their help in so many ways.

Every institution that could take soldiers has done so. In October the Royal Infirmary placed over two hundred beds at the disposal of the military authorities, and three weeks ago added one hundred more, in all about twelve hundred soldiers have been nursed there. Eighteen Red Cross nurses are working in the wards.

In September, Worsley Hall, lent by the Earl of Ellesmere, became a hospital; with 130 beds, twenty-seven nurses, and two resident surgical officers it has been possible to do much; and the soldiers there must find the beautiful grounds and surroundings a great incentive to recovery. They remain until almost well, when those unable to go home on furlough are sent to Southport for a time.

Week by week schools, etc., have been transformed into Voluntary Aid Detachment hospitals, and at the present time more than twenty are being occupied in this way. Some have 150 beds, in others there are not so many; in all there are hospital trained sisters, assisted by Red Cross nurses and orderlies. Those who cannot help in the actual work make easier the tedium of convalescence by many generous gifts.

A number of doctors have come to the assistance of those hospitals that have lost members of their staff, several of whom are serving at the Front and others at the military hospitals here.

Soldiers from practically every battle fought arrive: from Mons in the early days, from Ypres, Australians from the Dardanelles, Canadians, men who have won the V.C., Belgian officers, British officers; day after day they come, three thousand in one week not long ago.

When this great war is at an end the people of Manchester will have given many men to their country, and will have saved many.

